# Dr. Samuel Max Brickner

**Published:** 1916-06

**Authors:** 


**Dr. Samuel Max Brickner,** P. & S. 1891, died at Saranac Lake, May 5. He was born in Rochester in 1867, graduated at the University of Rochester 1888, practiced gynaecology in New York City until about two years ago when he was compelled to seek a better climate on account of his health. He was Associate Editor of the N. Y. Medical Journal and had recently established the Medical Pickwick. A more complete obituary will be published later.

